# Eicosapentaenoic acid ethyl ester improves endothelial dysfunction in type 2 diabetic mice

**DOI:** 10.1186/s12944-018-0770-0

**Published:** 2018-05-22

**Authors:** Yasuhiro Takenouchi, Kazuo Ohtake, Koji Nobe, Keizo Kasono

**Affiliations:** 1 Department of Pharmacology, Kawasaki Medical School, 577, Matsushima, Kurashiki, Okayama 701-0192 Japan; 2 Laboratory of Physiology, Faculty of Pharmaceutical Sciences, Josai University, Sakado, Saitama, 350-0295 Japan; 3 Division of Pharmacology, Department of Pharmacology, Toxicology Therapeutics, School of Pharmacy, Showa University, Shinagawa-ku, Tokyo, 142-8555 Japan

**Keywords:** Diabetes, Endothelium, Eicosapentaenoic acid, Thoracic aorta

## Abstract

**Background:**

Eicosapentaenoic acid (EPA) is thought to have many beneficial effects, such as anti-atherosclerogenic and anti-inflammatory properties. However, few studies have reported its effects of endothelial dysfunction in diabetes and its direct effects on the aorta. Here, we investigated the effects of EPA treatment on impaired endothelium-dependent relaxation of the aorta in KKAy mice, a model of type 2 diabetes.

**Methods:**

Male KKAy mice were fed a high-fat (HF) diet for 8 weeks to induce diabetes, after which they were divided into two groups. One group was fed a HF diet, and the other group was fed a HF diet containing EPA ethyl ester (EPA-E, 10 mg/day) for 4 weeks. Then, the vascular reactivities of prepared aortic rings were measured in an organ bath to determine if EPA-E administration changed vascular function in these diabetic mice. In addition, we examined effect of EPA-E and its metabolites to vascular action using aorta separated from C57BL/6 J mice.

**Results:**

Although EPA-E administration did not change the plasma glucose and insulin levels in diabetic mice, total cholesterol levels were significantly decreased. The aorta extracted from EPA-E untreated diabetic mice showed impaired endothelium-dependent relaxation in response to acetylcholine (ACh). However, EPA-E administration improved the relaxation response to ACh to the control levels observed in non-diabetic C57BL/6 J mice. On the other hand, endothelium-independent relaxation in response to sodium nitroprusside did not significantly differ among these three groups. The enhanced contractile response by phenylephrine in diabetic mice was not altered by the administration of EPA-E. In addition, the direct administration of EPA-E metabolites such as EPA, docosahexaenoic acid, and docosapentaenoic acid led to vasodilation in the aortic rings of C57BL/6 J mice.

**Conclusion:**

These results showed that chronic EPA-E administration prevented the development of endothelial dysfunction in KKAy mice, partly via the direct action of EPA-E metabolites on the aorta.

## Background

Eicosapentaenoic acid (EPA) is an *n*-3 polyunsaturated fatty acid (*n*-3 PUFA) that is abundant in fish oils. Epidemiological and clinical trials have shown that *n*-3 PUFA, including EPA, reduces cardiovascular disease risk [1–3] and delays the progression of atherosclerosis in patients with coronary disease [4]. Many studies have demonstrated that *n*-3 PUFAs have a variety of bioactive actions such as anti-inflammatory properties [5, 6], antioxidant effects [7, 8], and improvement of endothelial function [9, 10], which explains their anti-atherogenic effects. In a previous experiment, we reported that fish oil feeding combined with food restriction improved dyslipidemia and serum levels of adiponectin [11] and decreased lipid contents in the liver [12]. However, the effects of EPA-associated cardiovascular protection in diabetes mellitus are not completely understood.

Type 2 diabetes mellitus (T2DM) is a dominant risk factor for the development and progression of atherosclerosis. Usually, endothelial dysfunction precedes the onset of atherosclerosis and occurrence of cardiovascular complications, and patients with long-term T2DM have pronounced endothelial dysfunction, leading to increased cardiovascular disease risk [13, 14]. Endothelial dysfunction is defined as reduced endothelium-dependent vasodilator function, and decreased levels of bioavailable nitric oxide (NO) in the arteries. It has been suggested that the improvement of endothelial dysfunction prevents atherosclerosis [15]. KKAy mice develop obesity, elevated plasma glucose, and insulin resistance, all of which are characteristics of T2DM [16, 17].

In the present study, we investigated the effects of EPA on the aorta of KKAy mice fed a high-fat (HF) diet.

## Methods

### Reagents

EPA, docosapentaenoic acid (DPA), docosahexaenoic acid (DHA), N-ω-nitro-L-arginine (L-NNA), and phenylephrine hydrochloride were purchased from Sigma Chemical Co. (St. Louis, MO, USA). Sodium nitroprusside dehydrate (SNP) was from Wako Chemical Company (Osaka, Japan). Acetylcholine chloride (ACh) was from Daiichi Pharmaceuticals (Tokyo, Japan). Eicosapentaenoic acid ethyl ester (EPA-E) was from Tokyo Chemistry Industry (Tokyo, Japan). EPA, DPA, and DHA were dissolved in methanol, and the other reagents were dissolved in saline. All concentrations are expressed as the final molar concentration in the organ bath.

### Experimental design

Male KKAy and C57BL/6 J mice were obtained from Tokyo Laboratory Animals Science (Tokyo, Japan) at 6 weeks of age and fed a standard pelleted diet (CE2; CLEA, Tokyo, Japan) for 1 week. Mice were exposed to a 12-h light–dark cycle and maintained at a constant temperature of 22 ± 2 °C and humidity of 55 ± 10%. KKAy mice were fed a HF diet consisting of 38.9 energy% lard oil as the fat source for 8 weeks. These mice with HF diet-induced obesity were subsequently divided into two groups (*n* = 6 in each group). For another 4 weeks, one group was fed a HF diet (KKAy-HF), and the other group was fed a HF diet containing EPA-E (10 mg/day; KKAy-HF + EPA-E). C57BL/6 J mice were fed a Normal diet. These Normal, HF and HF + EPA diets were made based on the modified version of the AIN-93G [18]. The compositions of these diets are indicated in Table 1. The mice used for the experiments of Fig. 4 were C57BL/6 J male mice fed a CE2 at 6–8 weeks of age. The animal experiments were approved by the Institutional Animal Care and Use Committee of Josai University (Saitama, Japan).

**Table 1:** Compositions of experimental diets

Ingredients	Normal	HF	HF + EPA-E
Corn starch (g)	479.5	339.5	339.5
Sucrose (g)	150	150	150
Casein (g)	200	200	200
L-Cystein (g)	3	3	3
Cellulose (g)	50	50	50
Soybean oil (g)	40	40	40
Lard (g)	30	160	158
Cholesterol (g)	0	10	10
Mineral mix (AIN-93G) (g)	35	35	35
Vitamin mix (AIN-93G) (g)	10	10	10
Choline bitartrate (g)	2.5	2.5	2.5
Tert-Butylhydroquinone (g)	0.01	0.01	0.01
Eicosapentaenoic ethyl ester (g)	0	0	2
Total (g)	1000	1000	1000
kcal	4159.9	4859.9	4859.9
Fat % (Calories)	15.1	38.9	38.9

### Measurement of plasma levels of glucose, insulin, and cholesterol

Plasma parameters were measured as previously described [19]. Blood samples were centrifuged (1000 g for 20 min at 4 °C), and the plasma was stored at −20 °C until subsequent assays. Briefly, plasma levels of glucose, triglyceride and total cholesterol were determined with each commercially available enzyme kit (Wako Chemical Company, Osaka, Japan). Plasma insulin was measured by enzyme-linked immunoassay kit (Shibayagi, Gunma, Japan).

### Measurement of isometric force

Each aorta was separated from the surrounding connective tissue and cut into rings of 3 mm long, as previously described [19–21]. For the vasorelaxation studies, aortic rings were precontracted with an equieffective concentration of prostaglandin F_2_α (PGF_2_α) (1 × 10^− 6^–3 × 10^− 6^ M). When the PGF_2_α-induced contraction had reached a plateau level, ACh (10^− 9^–10^− 5^ M) or SNP (10^− 10^–10^− 5^ M) was added in a cumulative manner, then EPA (10^− 5^ M), DPA (10^− 5^ M), or DHA (10^− 5^ M) were administered in a single dose. To elucidate the effects of NO, a NO synthase (NOS) inhibitor (L-NNA) (10^− 4^ M) was added to the bath 30 min before vascular contraction reaction in the same experiments.

### Statistical analysis

All results are expressed as the mean ± standard error of the mean (SEM). Plasma parameters and body weight were compared by analysis of variance (ANOVA) followed by Scheffe’s post hoc test. Statistical comparisons of concentration–response curves were performed using two-way ANOVA with the Bonferroni post hoc test to correct for multiple comparisons. These statistical analyses were performed using the StatView program (SAS institute, Cary, NC, USA). A *P* value < 0.05 was deemed statistically significant.

## Results

### Plasma levels of glucose, insulin, cholesterol, and triglyceride and body weight

**Fig. 1:**
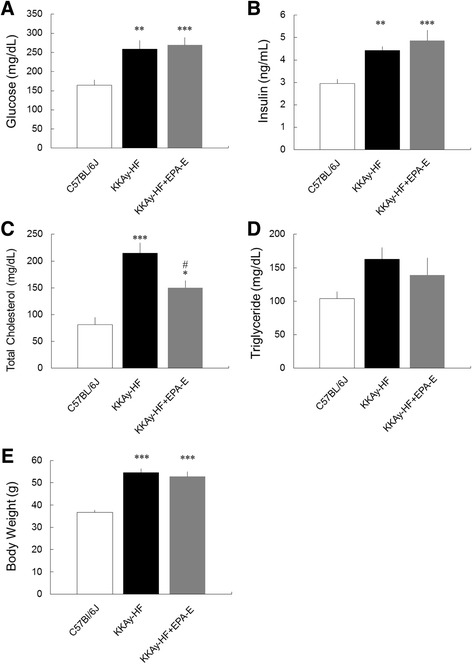
Plasma glucose (**a**), insulin (**b**), total cholesterol (**c**), and triglyceride (**d**) levels and body weight (**e**) in the three experimental groups. The increase in total plasma levels of cholesterol was significantly decreased by the administration of EPA-E (**c**). However, other parameters were not affected by the administration of EPA-E (**a**, **b**, **d**, **e**). Data are expressed as mean ± SEM for eight mice. *P < 0.05 **P < 0.01, ***P < 0.001 vs. C57BL/6 J, #*P* < 0.05 ##*P* < 0.01 vs. KKAy-HF mice

As shown in Fig. 1, non-fasting plasma levels of glucose, insulin, and total cholesterol and body weight were significantly elevated in KKAy-HF diabetic mice compared to age-matched C57BL/6 J mice. The increase in total cholesterol was decreased by administration of EPA-E for 4 weeks (Fig. 1c). However, plasma levels of glucose and insulin and body weight were not affected by EPA-E (Fig. 1a, b, e). In addition, plasma levels of triglycerides were not significantly different among the three groups (Fig. 1d).

### Vascular reactivity in aorta

**Fig. 2:**
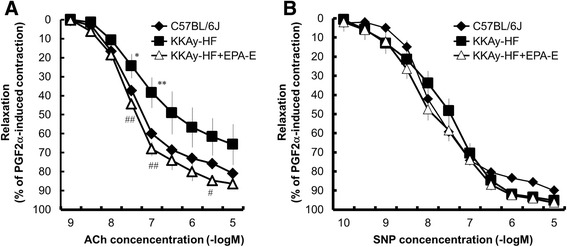
Concentration–response curves following ACh (**a**) and SNP (**b**) treatments of mice aortic rings. **a** Compared to C57BL/6 J mice, ACh-induced relaxation of aortic rings extracted from KKAy-HF mice was significantly weaker. This attenuated relaxation was significantly improved by chronic EPA-E treatment. **b** The relaxation induced by SNP was not significantly different among the three groups. Data are expressed as mean ± SEM; *n* = 6; *P < 0.05 **P < 0.01 vs. C57BL/6 J, #*P* < 0.05 ##*P* < 0.01 vs. KKAy-HF mice

**Fig. 3:**
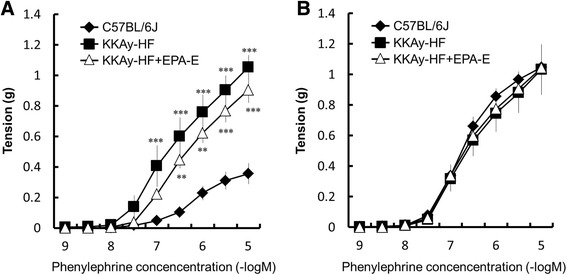
Concentration–response curves of phenylephrine-induced contraction of aortic rings (**a**) and contraction in the presence of L-NNA (**b**). **a** The increase in contractions in KKAy-HF mice was not affected by chronic administration of EPA-E. **b** In the presence of L-NNA, the contractions induced by phenylephrine were increased in C57BL/6 J mice and reached levels found in KKAy-HF and KKAy-HF + EPA-E mice. Data are expressed as mean ± SEM; *n* = 6–8; **P < 0.01 ***P < 0.001 vs. C57BL/6 J

**Fig. 4:**
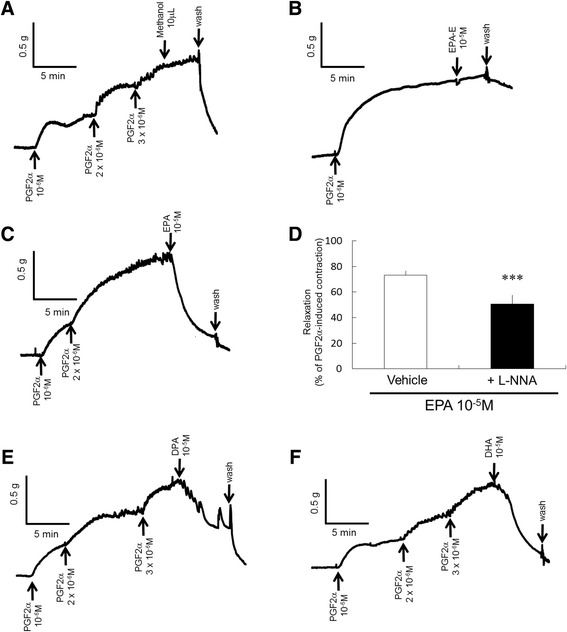
Effects of EPA-E, the metabolites and the solvent, methanol on the aortic rings extracted from C57BL/6 J mice in vitro. Original tracings showing that these reagents stimulated relaxation of the aorta after precontraction with prostaglandin F_2_α. EPA-E and methanol (a solvent of all fatty acids) did not relax the C57BL/6 J aortic rings (**a**, **b**). The EPA-E metabolites EPA, DPA, and DHA induced vasodilation in the aortic rings (**c**, **e**, **f**). In the presence of L-NNA, the relaxation induced by EPA was decreased in the aortic rings (**d**). Data are expressed as mean ± SEM; *n* = 8; ***P < 0.001 vs. Vehicle

The ACh-induced relaxation of aortic rings extracted from KKAy mice was significantly weaker than that of C57BL/6 J mice (Fig. 2a). This attenuated relaxation was significantly improved by chronic EPA-E treatment (Fig. 2a). The relaxation induced by SNP was not significantly different among the three groups (Fig. 2b). Phenylephrine caused concentration-dependent contractions that reached a maximum at 10^− 5^ M in aortic rings. Phenylephrine-induced contraction was significantly greater in KKAy-HF mice than in C57BL/6 J mice (Fig. 3a). The increase in contraction in KKAy-HF mice was not affected by the chronic administration of EPA-E (Fig. 3a). Next, the effects of a NOS inhibitor (L-NNA) (10^− 5^ M) on the response to phenylephrine were examined. The contraction induced by phenylephrine was increased in C57BL/6 J mice and reached the levels observed in KKAy-HF and KKAy-HF + EPA-E mice (Fig. 3b). Then we observed the direct actions of EPA-E and its metabolites on the aorta. When each metabolite was administered to the organ bath in a single dose, as shown by the representative tracings in Fig. 4a and b, EPA-E and methanol (a solvent of all fatty acids) did not relax the C57BL/6 J aortic ring precontraction with PGF_2_α. Interestingly, EPA, DPA, and DHA induced vasodilation in the aortic rings (Fig. 4c, e, f). Furthermore, the vasodilation induced by EPA was suppressed in the presence of L-NNA (Fig. 4d).

## Discussion

The results of the present study showed that chronic dietary EPA-E supplementation improved the endothelial dysfunction seen in type 2 diabetic mice fed a HF diet. Furthermore, EPA-E metabolites (including EPA, DHA, and DPA), but not EPA-E itself, had beneficial effects on vascular function in mice aortic rings.

Metabolic disorders including obesity, hyperglycemia, dyslipidemia, and hyperinsulinemia were seen in KKAy mice (Fig. 1), indicating that these mice are suitable models for T2DM with obesity. Among these unusual parameters, the increase in total plasma levels of cholesterol was significantly decreased by the chronic administration of EPA-E.

Although the measurement of flow-mediated dilation (FMD) is a standard method for measuring endothelial function in humans, the measurement of isolated vascular reactivity in the organ bath is used in animals because of technical limitations of doing these experiments in mice [22]. To examine the endothelium-dependent and endothelium-independent relaxation of the isolated aorta, we used ACh and SNP (Fig. 2). We found that the ACh-induced endothelium-dependent relaxation was impaired in KKAy-HF compared with wild type C57BL/6 J mice (Fig. 2a). Because the endothelium-independent relaxation of aortic rings induced by SNP was not different between C57BL/6 J and KKAy-HF mice (Fig. 2b), the activity of soluble guanylate cyclase in the smooth muscle of the aorta was not altered in KKAy-HF mice. Clinical studies have demonstrated that endothelial impairment in diabetes is the initial step of atherosclerosis [3]. Our results indicated that EPA-E can effectively improve endothelial function in the thoracic aortas of diabetic mice. Previous FMD experiments demonstrated that *n-3* PUFA (including EPA and DHA) supplementation improved vascular function or coronary vasomotion [23–25]. However, pure EPA in the diet is very unstable and very easily oxidized. Therefore, the more stable ethyl-esters EPA is usually administered to patients in clinical studies [26]. Thus, it is possible that EPA-E administration reduces the progression of atherosclerotic disorders in humans.

In a previous report, dietary supplementation with fish oil or EPA was shown to reduce contractile responses evoked by noradrenaline and arachidonic acid in the rat aorta [27]. However, the enhanced contractile response by phenylephrine in KKAy-HF was not altered by the administration of EPA-E in our experiments, and contractions induced by phenylephrine in the presence of L-NNA did not significantly differ among the three groups (Fig. 3b). Phenylephrine, an α-adrenergic receptor agonist, stimulates Ca^2+^ entry through voltage-gated and store-operated Ca^2+^ channels [28], and activates Ca^2+^-sensitization pathways such as protein kinase C (PKC) and Rho-kinase [29, 30]. NO reduces Ca^2+^ entry into vascular smooth muscle [31] and c-GMP-dependent protein kinase causes phosphorylation and inactivation of myosin light-chain kinase leading to inactivation of Ca^2+^-sensitive pathways such as PKC and Rho-kinase [29]. In these experiments, although chronic EPA-E administration improved endothelial dysfunction in KKAy-HF mice, the vascular contractile response in KKAy-HF + EPA-E mice showed no change compared with KKAy-HF mice. Further investigations on the vascular contractile response are needed.

EPA-E administration did not change plasma glucose or insulin levels in our experiments. Our data showed that chronic EPA-E administration improved endothelial dysfunction independently of glucose homeostasis. It has been speculated that the vascular protective effects of EPA-E administration are attributable, at least in part, to the enhanced endothelial function of vasoprotective factors such as endothelium-derived relaxation factor including NO and prostacyclin. To clarify the vasoprotective effects of EPA-E, the relaxation of aortic rings following the direct administration of EPA-E in an organ bath was observed. Despite the beneficial effects of endothelial function in a previous in vivo study, EPA-E did not directly affect the vascular reaction. It has been postulated that the improvement of endothelial dysfunction in type 2 diabetic mice by chronic EPA-E administration is partly due to a decrease in total cholesterol levels and anti-platelet actions [32]. In addition, we speculated that EPA-E metabolites may have vasoprotective effects. In an EPA-E metabolism study, it was reported that when labeled ^14^C-EPA-E was orally administered to rats, EPA, DPA and DHA were identified as metabolites of EPA-E [33]. In this study, we found that these metabolites directly affected vascular reactivity.

Our present observations clarified that EPA, DPA, and DHA as EPA-E metabolites caused vasodilation in C57BL/6 J mice aortic rings. It has been reported that EPA induces vascular relaxation in both endothelium-dependent [10] and endothelium-independent [34] manners. Here, we found that EPA-induced relaxation partly occurred via eNOS activation because relaxation was attenuated in the presence of a NOS inhibitor. Further investigations are needed to clarify the mechanisms underlying vascular relaxation by these EPA-E metabolites.

## Conclusions

We demonstrated that impairment of endothelium-dependent relaxation in aortas from type 2 diabetic mice was prevented by chronic EPA-E administration partly by the direct vasodilative action of EPA-E metabolites.

## Abbreviations

ACh: Acetylcholine
ANOVA: Analysis of variance
DHA: Docosahexaenoic acid
DPA: Docosapentaenoic acid
EPA: Eicosapentaenoic acid
EPA-E: Eicosapentaenoic acid ethyl ester
HF: High fat
L-NNA: N-ω-nitro-L-arginine
NO: Nitric oxide
NOS: Nitric oxide synthase
PGF_2_α: Prostaglandin F_2_α
PKC: Protein kinase C
SEM: Standard error of the mean
SNP: Sodium nitroprusside
